# Evaluation and pharmacovigilance of projects promoting cultivation and local use of *Artemisia annua *for malaria

**DOI:** 10.1186/1475-2875-10-84

**Published:** 2011-04-11

**Authors:** Merlin L Willcox, Shelly Burton, Rosalia Oyweka, Rehema Namyalo, Simon Challand, Keith Lindsey

**Affiliations:** 1 Department of Primary Health Care, University of Oxford, UK; 2 Research Initiative for Traditional Antimalarial Methods, Oxford, UK; 3 Department of Medical Anthropology, University of Oxford, UK; 4 Rural Extension for Africa's Poor (REAP), PO Box 6173, Kondele, Kisumu, Kenya; 5 Anamed Uganda, Masaka, Uganda; 6 Anamed International, Winnenden, Germany

## Abstract

**Background:**

Several non-governmental organisations (NGOs) are promoting the use of *Artemisia annua *teas as a home-based treatment for malaria in situations where conventional treatments are not available. There has been controversy about the effectiveness and safety of this approach, but no pharmacovigilance studies or evaluations have been published to date.

**Method:**

A questionnaire about the cultivation of *A. annua*, treatment of patients, and side-effects observed, was sent to partners of the NGO Anamed in Kenya and Uganda. Some of the respondents were then selected purposively for more in-depth semi-structured interviews.

**Results:**

Eighteen partners in Kenya and 21 in Uganda responded. 49% reported difficulties in growing the plant, mainly due to drought. Overall about 3,000 cases of presumed malaria had been treated with *A. annua *teas in the previous year, of which about 250 were in children and 54 were in women in the first trimester of pregnancy. The commonest problem observed in children was poor compliance due to the bitter taste, which was improved by the addition of sugar or honey. Two miscarriages were reported in pregnant patients. Only four respondents reported side-effects in other patients, the commonest of which was vomiting. 51% of respondents had started using *A. annua *tea to treat illnesses other than malaria.

**Conclusions:**

Local cultivation and preparation of *A. annua *are feasible where growing conditions are appropriate. Few adverse events were reported even in children and pregnant women. Where ACT is in short supply, it would make sense to save it for young children, while using *A. annua *infusions to treat older patients who are at lower risk. An ongoing pharmacovigilance system is needed to facilitate reporting of any adverse events.

## Background

*Artemisia annua *has been used as a herbal medicine for intermittent fevers since at least 340AD[1]. Artemisinin was isolated from *A. annua *in 1971 and is today the most powerful anti-malarial drug ever developed. Artemisinin combination therapy (ACT) is now recommended by WHO as the first-line treatment for uncomplicated malaria[2]. However, ACT remains relatively expensive and is not consistently available in many malarious areas, leading some NGOs to promote the use of herbal preparations of *A. annua*. Preliminary clinical trials suggest these can be effective even though the artemisinin content is low[3,4].

There has been much debate about the appropriateness, effectiveness and safety of such programmes[5]. Some critics believe it is too complicated to teach villagers how to grow, harvest and prepare a plant for their own use as a herbal medicine. Others fear that such practices may be ineffective and promote the spread of resistance to artemisinin, as the dose of artemisinin in the tea is lower than in tablets[6]. Conversely, some NGOs argue that these programmes are often the only means of providing a sustainable supply of anti-malarial medicine in remote areas, which have little or no healthcare infrastructure. Furthermore, they argue that the herbal medicine contains several anti-malarial compounds as well as artemisinin, which act synergistically and may prevent the development of resistance[7-10].

Since 1998 the NGO Action for Natural Medicine[11] has distributed 1,200 *A. annua *"starter-kits" (containing seeds and instructions for their use) to partners in 75 countries. In addition, Anamed key workers have run over 100 week-long training seminars on natural medicine in 20 different countries, mostly in Africa. Local people trained by Anamed continue to run week-long training seminars themselves. These seminars devote a lot of attention to the cultivation and use of *A. annua *and the full details are available in Anamed publications [12,13]. The preparation recommended is an infusion of 5 g of dried *A. annua *leaf powder in 1 litre of water, to be taken as 250 ml four times a day (for adults).

The aim of this study was to evaluate some Anamed projects promoting the cultivation and local use of *A. annua *for the treatment of malaria in East Africa. In particular, the focus was to examine the feasibility of setting up a pharmacovigilance system for potential adverse effects, including in pregnant women.

## Methods

A questionnaire was designed about the cultivation of *A. annua*, treatment of patients with malaria and other illnesses, and side-effects observed. Key partners in Kenya and Uganda were identified by Anamed International (as people who had previously participated in their seminars), and these partners in turn identified other possible respondents in their areas. Questionnaires were sent and completed electronically or through the local network of partners. Some of the respondents were then selected purposively for conducting more in-depth semi-structured interviews and participant observation. The selection prioritised those partners who had treated the most patients (in order to get as much information as possible about adverse events observed) and those whom it was feasible to visit within the time available. A local person interpreted during the interviews when necessary, although most respondents spoke good English.

## Results

In total, about 45 partners were contacted of whom 39 responded (87% response rate) by completing a questionnaire and/or an interview, between July 2009 and January 2011. In Kenya (Kisumu area), nine partners were visited and interviewed (in November 2009), and a further nine just filled out a questionnaire. In Uganda, fourteen partners were interviewed (Kampala, Makondo and Mbarara, July 2009, by SB; and Kasese and Bushenyi, January 2011, by MW) and a further seven just completed a questionnaire.

**Figure 1:**
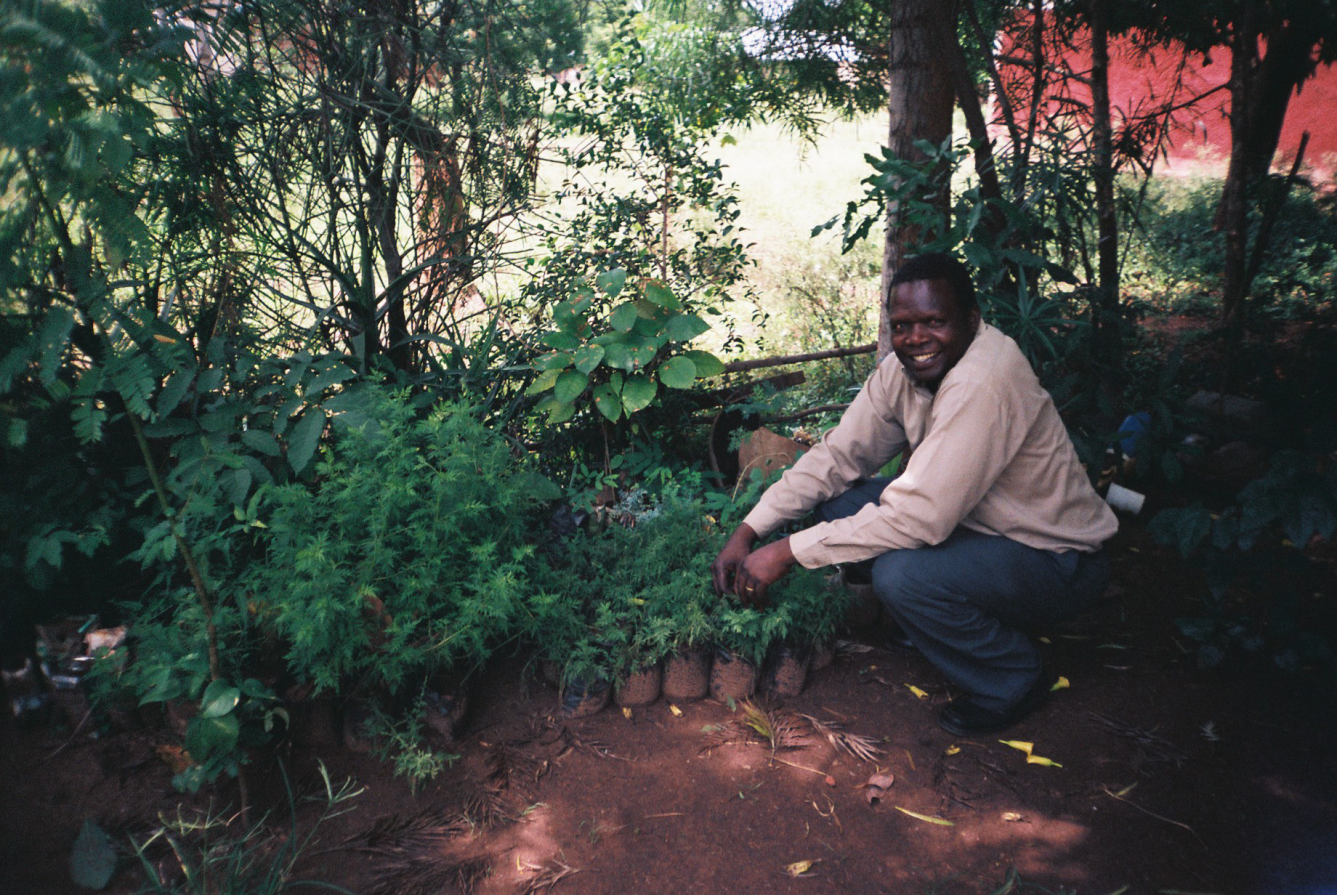
**Pastor George Okuto (Kisumu, Kenya) has distributed *Artemisia annua *plants to over 200 people in his community**.

**Figure 2:**
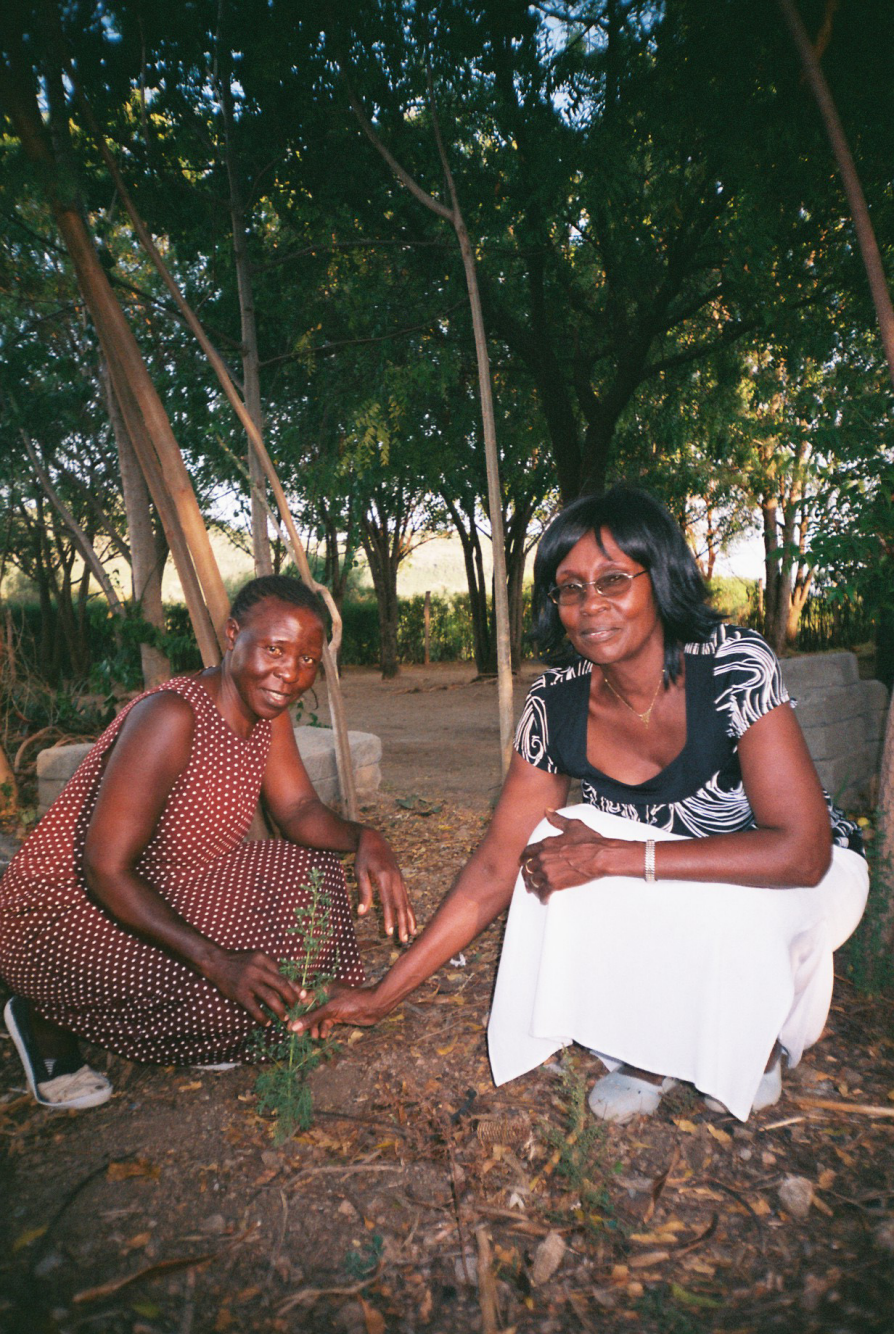
**Ester Odhiambo (Rusinga Island, Kenya) demonstrates poor growth of *Artemisia annua *due to drought**.

**Figure 3:**
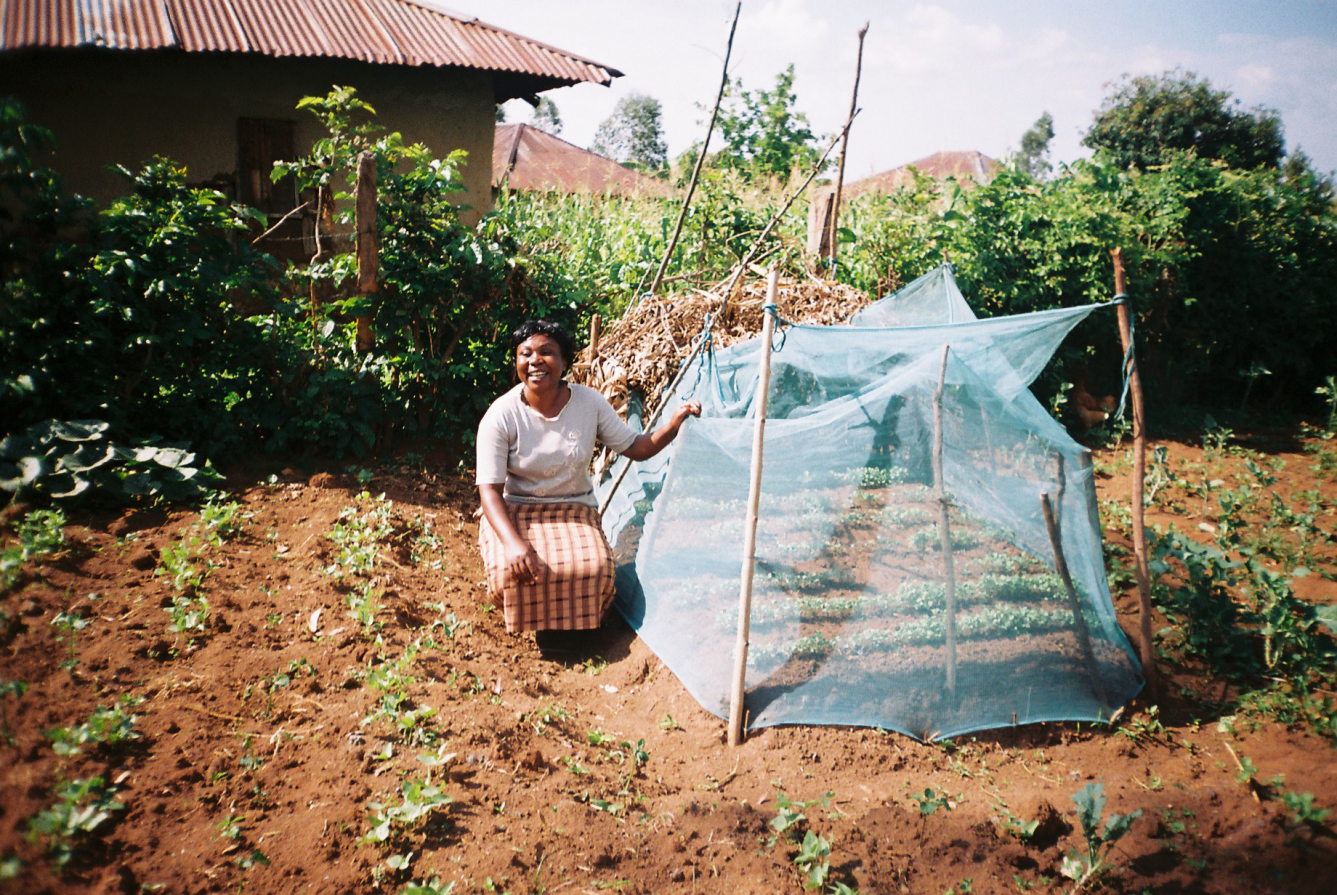
**Monica Khagoni (Ingidi, Kenya) with her mosquito-net shelter to protect *Artemisia annua *seedlings from hailstones**.

The demographic characteristics of the respondents are summarised in table 1. Thirteen of the respondents were health workers (equally split between biomedical and traditional). The majority of the respondents were not primarily health workers, but had pastoral roles in their communities (as priests, teachers and NGO workers) or simply as community members (farmers and mothers). In Kenya, *A. annua *has been disseminated primarily through the church network by Rural Extension for Africa's Poor (REAP) [14,15] (Figure 1). They had grown *A. annua *for an average of 3.3 years (range: <1 year to 8 years). Ten of the respondents grew it in a community garden, while all the others were growing it in their home gardens. Nineteen respondents (49%) mentioned they had had difficulty growing the plant. The commonest reason was drought (13 respondents) which caused the plants to die or flower too early (Figure 2). Two respondents mentioned difficulty in getting the seeds to germinate, and one mentioned that seedlings had been killed by hailstones. She had since developed a new system of protecting them - with a mosquito net (Figure 3)! Those who had no problems in cultivation tended to be more in highland areas with good rainfall.

**Table 1:** Characteristics of respondents

		N (%)
Sex	Female	21 (54%)
	
	Male	18 (46%)

Occupation	Minister of Religion (pastor/nun)	9 (23%)
	
	Manual worker (farmer/mother/builder)	8 (21%)
	
	Biomedical health worker (doctor/nurse/midwife/laboratory technician)	7 (18%)
	
	Healer/naturopath	6 (15%)
	
	Teacher	5 (13%)
	
	NGO worker	2 (5%)
	
	Other	2 (5%)

**Figure 4:**
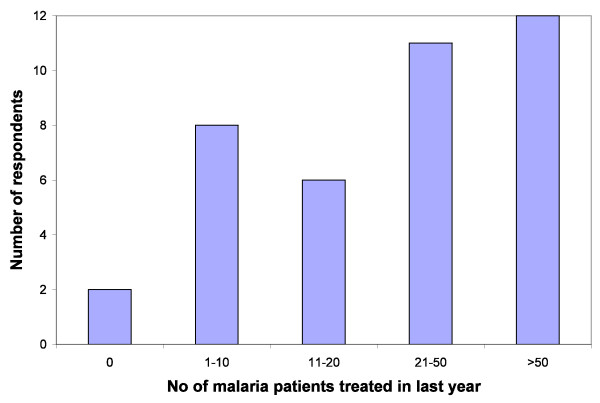
**Number of patients treated for presumed malaria with *Artemisia annua *infusion in the last year by each respondent**.

Figure 4 shows the number of patients treated for presumed malaria by each respondent in the last year; overall this adds up to approximately 3,000 patients. One respondent had not treated any patients in the previous year because the *A. annua *crop had failed due to drought. Most respondents fell into two broad categories: those using it mainly for themselves and their family (who, therefore, had treated less than 10 patients in the course of the year) and those who were treating many other people in their community. The latter included clerics, farmers, and health workers. One healer claimed she had treated three patients a day (which adds up to >1,000 over the year). Five others said they had treated over 100 malaria patients with *A. annua *in the last year. The vast majority said they gave the infusion for seven days, although three gave less (2-5 days) and one gave more (up to 10 days). One respondent used the infusion in combination with sulphadoxine-pyrimethamine (SP), but all others used it alone for the treatment of presumed malaria.

25 of the respondents (64%) said they had treated children under the age of five years with *A. annua *tea. Most had only treated small numbers (usually their own children) but four respondents had treated over 30 children in the past year with *A. annua *infusion; overall the number of children treated was at least 250. Most of these lived in fairly remote communities with no health centre. They reported that all of the children treated had recovered completely (although there was no formal system for follow-up). Three respondents mentioned that the bitter taste affects compliance, and eight reported that they mix the infusion with sugar or honey to improve the taste. Two respondents mentioned that the infusion seemed to be less effective in children, with a higher relapse rate (one mentioned that all the children under the age of three years had had a recrudescence). Only one respondent mentioned a rash in one child after taking the infusion, which settled after four days, while he was still taking the infusion. He had taken the infusion many times previously with no problems. No other adverse events were reported in children.

Eleven respondents (28%) had treated women in the first trimester of pregnancy with *A. annua *tea, and reported on a total of at least 54 patients. Two miscarried, one reported severe vomiting and one reported joint pains, weakness and pruritus. The other 50 women were reported to have had no adverse effects, and indeed most had already delivered a healthy baby (a few had not yet delivered). One doctor had conducted a "trial" of Artemisia tea for 20 women in the first trimester of pregnancy, and found that the remedy was better tolerated than quinine, so that he felt compliance and outcomes were better. He had followed up the pregnant women carefully with blood tests and ultrasound scans until their delivery, and all had delivered normal babies. He observed that some of the women continued taking the tea (against his advice) throughout their pregnancy as a prophylactic.

Although the questions focussed on the first trimester, five respondents mentioned that they had treated a total of nine pregnant women in their second trimester (in two cases they had treated themselves while pregnant). None of these had had any problems. Two respondents mentioned that women had started taking *A. annua *tea as a form of emergency contraception, or to induce abortion within one month of conception.

Apart from young children and pregnant women, only four respondents had observed adverse events in other patients. These were vomiting (three respondents), pruritus, rashes, diarrhoea and headache (each mentioned by one respondent). One respondent who was particularly careful about following up her patients (n = 17 over the last year) reported that about 20% reported a side-effect (one or more of vomiting, diarrhoea, rash).

Respondents were asked whether they had noticed the tea becoming less effective since they had started using it. Only five reported that they had, in a few patients, or that they now gave a longer treatment (ten days instead of seven).

Twenty respondents (51%) also mentioned that they had started using *A. annua *to treat illnesses other than malaria. These included: gastrointestinal conditions (pain/diarrhoea/typhoid/intestinal parasites - in 11 respondents), HIV/AIDS (11), haemorrhoids (5), cough (5), skin conditions (3), amenorrhoea (2), as a contraceptive (2), as an insect repellent (2), and various other miscellaneous uses.

## Discussion

Although many NGO projects promote the cultivation and local use of *Artemisia annua*, this is the first published evaluation of such a project. Although only a small sample of partners was interviewed, it is clear that local small-scale cultivation and preparation of *A. annua *are feasible in East Africa, within the context of broader development projects promoting self-reliance[14]. The church seems to have a better infrastructure than the formal health system, particularly in remote areas, and can reach communities which have no health services. The main practical problem was that cultivation was adversely affected by droughts in several areas. In drought-prone areas, drought-resistant anti-malarial plants such as *Argemone mexicana *would be more suitable[16-18]. *Artemisia annua *is more suited to highland areas with better rainfall.

One important caveat to this study is that it was retrospective, and it was not possible to test the patients to confirm whether they actually had malaria, or another cause for their symptoms. Nevertheless the areas selected have high transmission of malaria so it is reasonable to assume that a significant proportion of the cases reported did actually have malaria. Furthermore, the authors relied on the honesty of the respondents in reporting adverse events as they did not have the resources to track individual patients. Of course it is probable that not all adverse events were reported by patients to those who had treated them. This is a flaw inherent in almost all pharmacovigilance systems.

Although clinical trials of *A. annua *have all excluded children [5], almost two-thirds of respondents had used the infusion to treat malaria in young children (age <5 years) who are at greatest risk of severe malaria and death. The main problem observed was compliance, which can be improved by adding sugar or honey. Some had observed a higher relapse rate in young children. However in a situation where no other treatments are available, the infusion is probably better than nothing. Nevertheless clinical trials are needed to see whether the infusion can really be recommended for the treatment of young children.

Although ACT is now provided free of charge in government health facilities in many African countries, recent studies show that it was out of stock for 20% of the time in Kenya[19], and for 30% of patients in Uganda[20]. Interviews suggested that availability was even more limited than this in some areas. When health facilities had run out of ACT, Anamed partners were sometimes the only source of anti-malarial treatment in their community. In this situation of insufficient supply of ACT, it would make sense to save ACT for young children, while using *A. annua *infusions to treat older patients who are at lower risk (in high-transmission areas where residents develop partial immunity). The herbal infusion can also be combined with another anti-malarial drug which may be more readily available than an ACT. This practice was reported by two respondents: one only ever gave the infusion in combination with SP while another (a nurse) added SP in cases with high parasitaemia. All other respondents said they used *A. annua *infusion alone for the treatment of malaria.

Use of artemisinin in the first trimester of pregnancy is generally not recommended because of animal studies which have shown a potential to induce foetal resorption[21]. Artemisinin is generally considered to be safe in the second and third trimesters of pregnancy and now artesunate is recommended as the first line treatment for pregnant women with severe malaria[2]. Although Anamed does not recommend use of *A. annua *in the first trimester of pregnancy[12], sometimes this happens accidentally when a woman does not realize she is pregnant. On other occasions, *A. annua *tea may be given when no other treatments are available. Although some cases of miscarriage were reported after use of the plant, the incidence was relatively low (2/54 = 3.7%, 95% CI 0.5 - 12.7%) and is probably not greater than the background incidence in the population. This seems lower than the 20% miscarriage rate observed in 16 pregnant women who had taken artesunate in the first trimester[22] although the difference is not statistically significant (p = 0.13). It is possible that there may have been under-reporting of miscarriage in the first trimester. Very early miscarriages may not be viewed as abnormal or worth reporting. In Kenya abortion is illegal; one respondent was anxious that if it was found out that some of his patients had miscarried, he might be accused of procuring abortions. This fear may have deterred others from reporting miscarriages.

It was generally widely accepted that the infusion was well tolerated with few side-effects. Most of the "side-effects" reported could equally have been symptoms of malaria. For example it is difficult to determine whether nausea/vomiting was caused by the disease or the treatment. A randomised controlled trial has shown that *A. annua *infusion is better tolerated than quinine[4]. Nevertheless it is good practice to have a system of pharmacovigilance for any treatment, therefore we left forms with project partners to encourage them to report in detail any adverse events observed. These can be fed back via local networks (for example REAP) to Anamed. From now on Anamed will send feedback and pharmacovigilance forms to its partners one year after providing a "starter pack", to improve reporting of any adverse events. Setting up pharmacovigilance systems is also a challenge for biomedical treatments [23].

It is difficult to design a study to show whether use of Artemisia tea will promote resistance to artemisinin. There are arguments on both sides, but no proofs[5]. Although respondents had not been using the plant for very long, five reported that they thought it was becoming less effective. It would be interesting to examine this in more detail as there could be many causes. It is unlikely that the parasite would have evolved resistance to artemisinin over such a short period of time. A possible explanation is that high artemisinin yields in the standard hybrid plants will diminish in following generations if the plants are propagated by seeds rather than by vegetative means. Although Anamed promotes vegetative propagation (and this was practiced by many partners) some respondents did mention that the plants seed themselves, and they use the next generation of plants also. Even when using vegetative propagation as recommended, it has been found that artemisinin yields in some parts of East Africa are 0.20-0.35% (compared to the ideal of >1%)[24]. This may be due to different climatic conditions and lower altitude. In particular dry conditions will cause early flowering, which will reduce the yield of artemisinin (which is maximal just before flowering). For this reason it is important to develop a practical field method for quality control of *A. annua *plants.

Another interesting observation was that partners were using *A. annua *as a treatment for conditions other than malaria, especially diarrhoea/abdominal pain and HIV/AIDS. It is not impossible that the remedy is useful in these situations. Anamed has received informal feedback from many partners that they find *A. annua*, in combination with *Moringa oleifera *leaf powder, to be a useful supportive treatment in patients with HIV/AIDS[25]. Artemisinin has some antiviral activity against HIV itself and against opportunistic infections such as cytomegalovirus and herpes viruses[26]. *Artemisia annua *infusion has been shown to be active against faecal micro-organisms [27] and schistosomiasis[28], and also has an immunostimulatory effect on phagocytes in a mouse model[29]. Further research is needed to explore these possible beneficial effects.

## Conclusions

Local cultivation and preparation of *A. annua *are feasible where growing conditions are appropriate. Other plants may be better suited to drought-prone areas. Few adverse events were reported even in children and pregnant women, although further research is needed before deciding whether use of the remedy can be promoted in these groups. Meanwhile where ACT is in short supply, it would make sense to save it for young children, while using *A. annua *infusions to treat older patients who are at lower risk. A pharmacovigilance system is being set up to encourage reporting of any adverse events. *Artemisia annua *tea is also being used to treat other conditions, but further research is needed to determine whether it is effective for these.

## Competing interests

KL works for Anamed International, SC is voluntary coordinator of Anamed-UK and RN is coordinator of Anamed-Uganda. The other authors have no competing interests.

## Authors' contributions

MW, SB and KL designed the study, the questionnaire and interviews. KL, RO, RN and SC identified Anamed partners and distributed questionnaires. SB, MW, RO and RN visited participants and conducted interviews. MW drafted the manuscript and all authors read and approved the final manuscript.
